# Proteasome Inhibitors: Potential in Rheumatoid Arthritis Therapy?

**DOI:** 10.3390/ijms26072943

**Published:** 2025-03-24

**Authors:** Oktávia Tarjányi, Katalin Olasz, Fanni Rátky, György Sétáló, Ferenc Boldizsár

**Affiliations:** 1 Department of Medical Biology, Medical School, University of Pecs, H-7624 Pecs, Hungary; oktavia.tarjanyi@aok.pte.hu (O.T.); fanni.ratky@aok.pte.hu (F.R.); gyorgy.setalo.jr@aok.pte.hu (G.S.); 2 Department of Immunology and Biotechnology, Medical School, University of Pecs, H-7624 Pecs, Hungary; olasz.katalin@pte.hu

**Keywords:** proteasome inhibitors, rheumatoid arthritis therapy

## Abstract

Rheumatoid arthritis (RA) is a chronic autoimmune disease that leads to the destruction of peripheral joint cartilage and bone tissue. Despite the advent of biological therapies in the past decades, the complete remission of RA patients is still out of reach. Therefore, the search for novel therapeutic approaches is still open in the field of RA. Proteasome inhibitors (PIs) were originally designed to be used in hematological malignancies like multiple myeloma. However, evidence has shown that they are potent inhibitors of the NF-κB pathway, which plays a pivotal role in inflammatory processes and RA. Furthermore, inhibition of cell activation and induction of apoptosis was also reported about PIs. In the present review, we summarize the current knowledge about the potential effects of PIs in RA based on reports from animal and human studies. We believe that there is substantial potential in the use of PIs in RA therapy either alone or in combination with the medications already used.

## 1. Rheumatoid Arthritis

### 1.1. Pathogenesis

Rheumatoid arthritis (RA) is a systemic autoimmune disease affecting approximately 1% of the human population [1]. Progressive inflammation of the small synovial joints, with severe articular cartilage destruction leading to deformities and ankylosis, are characteristic of RA [1,2]. From the immunological point of view, RA is caused by a complex interplay of the innate (macrophages, neutrophils, pro-inflammatory cytokines, complement factors, etc.) and adaptive (T and B lymphocytes, Th1/2/17 cytokines, autoantibodies, etc.) immune mechanisms, and in the sequence of immunological events, the loss of tolerance due to perturbed immune regulation is of special importance [3]. RA is a polygenic disease with a special “constellation” of genetic components [4,5] that determine disease susceptibility and severity, modified by environmental factors [1,3,6], for example smoking (which promotes citrullination of self-proteins) [7], the composition of the oral and intestinal microbiome [8,9,10], and obesity [11]. Despite extensive studies, the triggering and/or causative factors of the disease are unknown; therefore, no causative treatment is available to date.

Pathological autoantibodies (ACPAs, anti-citrullinated protein antibodies), primarily targeting citrullinated self-proteins, can already be detected in the serum years before the clinical symptoms appear [2]. In this subclinical phase, tolerance mechanisms toward self-structures become impaired, allowing for the formation and synovial invasion of autoreactive T and B cells as well as antibodies [1]. The majority of ACPA-positive patients are also positive for rheumatoid factor (RF), which is associated with a significantly worse prognosis [12]. Two cartilage macromolecules of the extracellular matrix (type II collagen and proteoglycan (PG) aggrecan) have also been found as potential target autoantigens in RA [13]: the serum antibodies and T cells of RA patients react with their epitopes [14,15,16,17].

The synovium plays a crucial role in the pathogenesis of RA, acting as an ectopic tertiary lymphoid tissue housing cells involved the immune response (T and B cells) and tissue destruction [2,18,19,20]. The characteristic Th subpopulations in RA are Th1, Th17, Th22, and Treg, exhibiting considerable flexibility and plasticity in patients and producing significant amounts of inflammatory cytokines (IL-1, IL-6, IL-12, IL-17, and IFNγ), while Treg cells are responsible for controlling the pathological immune response by producing IL-10 and TGF-β [21,22].

### 1.2. Significance of the NF-κB Pathway in RA

An important signaling event in the inflammatory cascade of RA is the activation of the “canonical” NF-κB pathway in synoviocytes and immune cells. This pathway is activated by several receptors, such as IL-1-, TNF-, T-cell, and B-cell receptors and TLRs. Ligand binding to these receptors results in the phosphorylation, ubiquitination, and proteasomal degradation of IκB, releasing the previously inactive NF-κB for translocation into the nucleus, where it can bind to the promoter region of more than 150 responsive genes [23,24]. This results in the activation of various cell functions involved in inflammation, such as the release of TNFα from macrophages; production of MMPs [25,26] and cytokines by FLS; and the infiltration, activation, and proliferation of T cells, B cells, and neutrophils in the synovium. Osteoclast differentiation, proliferation, and activation is also controlled by NF-κB [27]. Experimental setups where the NF-κB pathway was suppressed by stable gene transfer of IκBα cDNA inhibited pro-inflammatory cytokine expression but had no effect on the production of the anti-inflammatory cytokine IL-10 [28,29]. The NF-κB pathway, in addition to the classical or “canonical” route, can also be activated via the “alternative” path. In patients with RA, this “alternative” mechanism is mainly triggered by the binding of B-cell-activating factor (BAFF) to the BAFF receptor (BAFF-R), which is associated with an overabundance of autoantibody-producing B cells [30]. BAFF antagonists such as belimumab (an anti-BAFF antibody) show promising results in the treatment of RA [31]. The NF-κB-inducing kinase (NIK), a crucial mediator of the “alternative” pathway, is also overexpressed in the synovium of RA patients, promoting pathological angiogenesis and synovitis [32]. Furthermore, NIK has been shown to play a significant role in experimental animal models; for example, NIK^−/−^ mice demonstrate a high degree of resistance to the development of arthritis [33]. Another critical cell type involved in tissue destruction in RA is the osteoclast. Osteoclast precursor cells are drawn into the RA synovium by the overproduction of NF-κB-dependent cytokines (IL-1β, TNFα, IL-6, and IL-17) [34]. High expression levels of receptor activator of nuclear factor-κB ligand (RANKL) (also known as TRANCE, TNF-related activation-induced cytokine, or ODF, osteoclast differentiation factor) in the inflamed synovium facilitate osteoclast differentiation, maturation, and bone resorption [35].

The significance of the NF-κB pathway in inflammation is notable, indicating substantial potential for its inhibition in treating autoimmune diseases such as RA [36]. Over the past few decades, numerous NF-κB inhibitors have been developed, including a variety of natural and synthetic agents such as antioxidants, proteasome inhibitors, peptides, small molecules, and dominant-negative or constitutively active polypeptides. Some of these compounds act as general inhibitors of NF-κB induction, while others specifically block certain induction pathways [37]. As the NF-κB pathway is not only activated in cells involved in inflammatory diseases, but it is also involved in a range of physiological functions, the true therapeutic potential lies in selectively blocking inflammatory cells. Our current understanding has largely emerged from transgenic and knockout animal models that have been instrumental in mapping and studying the NF-κB pathway [38,39]. Subsequent research has focused on the elements of this pathway in relation to immune functions, cancer, and inflammation. The administration of NF-κB decoys has been shown to reduce the severity of collagen-induced arthritis in rats [40]. Targeting the IκB kinase IKKβ, a mediator of NF-κB, can suppress the classical NF-κB pathway and inhibit osteoclast maturation and bone destruction [41]. Additionally, targeting NF-κB activation through protein phosphatases, methyltransferases, or p65 acetylation remains a topic of ongoing research [42].

Various approaches aim to impede the proteasomal degradation of IκB using proteasome inhibitors (for details, see Section 4). The first PI effectively used in human autoantibody-mediated autoimmune diseases was bortezomib [43,44]. Nonsteroidal anti-inflammatory drugs (NSAIDs), such as aspirin and ibuprofen, are designed to relieve pain and inflammation. Their mechanism of action involves inhibiting the synthesis of inflammatory mediators—e.g., prostaglandins—and, as inhibitors of the ATP binding site of IKK2, they can also prevent the phosphorylation of IκB, thereby inhibiting the translocation of NF-κB to the cell nucleus [45].

Keeping NF-κB bound in an inactive form reduces the expression of the adhesion molecules ICAM-1 and VCAM-1 on endothelial cells, thereby limiting lymphocyte and neutrophil extravasation into the synovium [46]. Glucocorticoids (GCs) such as dexamethasone, prednisone, and methylprednisolone are also first-line treatments for autoimmune diseases and are potent anti-inflammatory agents. GCs not only prevent NF-κB from binding to its target genes but also suppress the expression of those genes [47]. Both NSAIDs and GC medications may lead to serious side effects, including gastrointestinal ulcers and bleeding, liver and kidney dysfunction, increased BMI, infections, cardiovascular complications, etc. Thus, the benefits and risks of treatment and possible side effects must always be weighed against each patient’s individual condition [48].

### 1.3. Animal Models for RA

In addition to human studies, animal models of RA have added significantly to our knowledge of the disease pathomechanism and genetics because they represent the human disease in many ways [49]. Traditionally, the models are classified into two basic categories, spontaneous and inducible, corresponding to whether the injection of antigen(s) or adjuvants is necessary for their development or not. Herein, we describe some selected models briefly, paying special attention to those that have been used in testing the proteasome inhibitors, mentioned in Table 1. Autoimmune arthritis develops spontaneously in mice that carry spontaneous or genetically engineered mutations (e.g., SKG mice [50], human TNF transgenic mice (hTNF tg.) [51], PG-specific-TcR transgenic mice [52], or HLA-DR4 transgenic mice [53]). On the other hand, arthritis can be induced by injecting cartilage antigens into susceptible mice, like the abovementioned collagen (CIA) [54] or proteoglycan aggrecan (PGIA) [17] or the recombinant G1 domain of the aggrecan (GIA) [55]. In CIA, upon the injection of type II collagen with complete Freund’s adjuvant into susceptible mice or rats, progressive autoimmune inflammation of the small joints develops with autoantibody and inflammatory cytokine production [54]. Th17 dominance is typical for CIA [56]. Collagen antibody-induced arthritis (CAIA) is induced by the injection of a mixture of collagen-specific monoclonal antibodies with LPS [57]. Despite its obvious advantages (short induction time, can be used in any mouse strain), CAIA is not a real autoimmune arthritis; immunologically, it is considered rather as a passively transferred antibody-induced arthritis. Arthritis can also be effectively induced by the injection of various microbial products: complete Freund adjuvant-induced arthritis (AIA), containing Mycobacterial components [58], zymosan (a yeast glycan) injection into the knee joints [59], or Streptococcal cell wall-induced arthritis (SCWIA) [60]. In these latter models, activation of the innate immune system leads to the inflammation of the joints.

### 1.4. Current Therapeutic Approaches for RA

Overall, in the last two decades, there has been significant progress in the therapeutic possibilities in RA that led to slower disease progression and improved life quality of the patients; however, none of these offer a complete remission, and the termination of treatment almost certainly leads to the flare-up of the symptoms. The newer nomenclature of the anti-rheumatic drugs distinguishes the classical (“c”) and biological (“b”) disease-modifying anti-rheumatic drugs (DMARDs) [61]. Among cDMARDs, methotrexate is still the most frequently used drug, while the bDMARD group includes a wide range of monoclonal antibodies and biologically engineered molecules that target different molecular components of RA. The common clinical approach is to start the treatment with cDMARD, and in case of therapy resistance, switch to one of the bDMARDs.

Among the bDMARDs, TNFα inhibitors have been successfully employed in RA treatment for the past two decades. TNFα is elevated in the synovial joints of RA patients and is a key inflammatory cytokine that powerfully induces the NF-κB pathway [62]. TNFα antagonists, including infliximab (IFX), etanercept (ETN), adalimumab (ADA), golimumab (GOLI), and certolizumab pegol (CZP), have been widely used to treat RA. These TNF antagonists can reduce disease activity and slow joint destruction when used in monotherapy. They demonstrate similar efficacy but have distinct pharmacokinetic and -dynamic properties; combinatory therapy with methotrexate can further alleviate synovial inflammation and tissue damage. Side effects—including infections, adverse reactions, and injection-site reactions—may occur, although their incidence is low [63].

For RA patients who do not respond adequately to TNFα antagonists, rituximab may be considered as an alternative [64]. Rituximab is a chimeric monoclonal antibody targeting the CD20 molecule found on the surface of B cells, depleting pro-B cells and plasma cells. Total B-cell depletion through repeated rituximab treatments can achieve sustained efficacy in RA. Extended observation from randomized controlled trials has not shown a significant increase in serious event incidence, although repeated rituximab treatment is associated with hypogammaglobulinemia, which can elevate the risk of serious—but infrequent—opportunistic infections [64].

If TNFα antagonists prove ineffective, another therapeutic option is abatacept, a soluble fusion protein comprising the extracellular domain of human CTLA-4 and a fragment of the Fc portion of human IgG1 (hinge and CH2 and CH3 domains). Abatacept binds to human B7 (CD80/86) with higher affinity than CD28, thus inducing a blockade of T-cell co-stimulation, significantly reducing synovial inflammation and preventing bone and cartilage degradation [65].

Targeting the pleiotropic effects of IL-6, a crucial cytokine in RA, also offers an alternative to TNFα antagonists. The first approved humanized anti-IL-6 receptor monoclonal antibody was tocilizumab, followed by the development of newer IL-6 blockers such as sirukumab, olokizumab, clazakizumab, and sarilumab [66,67]. IL-6 blockers exhibit favorable efficacy and safety profiles, offering greater specificity for RA treatment compared with the broader effects of the TNF blockade [67,68].

Finally, Janus kinases (JAKs) mediate signaling through the IL-6 and other cytokine receptors; therefore, JAK inhibitors represent promising targets for the treatment of RA. JAK inhibitors, such as tofacitinib, baricitinib, and upadacitinib, are small-molecule therapeutics administered orally, providing a more favorable option compared with intravenously administered TNF antagonists [69].

The long list of medications above already suggests that none of them offer a complete treatment solution to all patients. The first problem is that the selection of the drugs is still based on a conventional stepwise clinical approach (i.e., when methotrexate is not effective, the next is TNF-blocking therapy, and when it fails, then rituximab/abatacept, and now the JAK inhibitors are tried). It is already evident from the complex pathogenesis of RA, and underlined by how many different ways arthritis can be induced in animal models, that there might be significant individual variations in what drives the disease, despite the common clinical symptoms. It is tempting to speculate about the possible benefits to test some more specific biological markers in RA patients before starting the treatments, to elucidate which of the cytokine(s) or which cell type(s) dominate their arthritis, to pick a more rationalistic personalized therapy. Second, despite the given treatment modalities, there still remains a group of patients who are therapy resistant, hence categorized into the “difficult-to-treat (D2T) RA” group lately [70]. Depending on the different clinical studies, the ratio of this group can vary between 5 and 27% of RA patients [71]. In the D2T group belong those patients where a minimum of 2 bDMARDs (from different groups) have proved ineffective [61,71]. For this latter group, it is essential that alternative medications targeting other pathways are introduced and tested. The third problem could be the side effects, which are also variable but in some cases, some medications must be stopped or replaced because of serious or intolerable adverse effects despite their efficacy. Based on these reasons, the testing of new treatment types in RA, for example, the proteasome inhibitors, is of significant clinical importance.

## 2. The Ubiquitin–Proteasome System (UPS)

All eukaryotic cells have a well-regulated way of breaking down unneeded, unfolded, misfolded or damaged proteins [72]. The ubiquitin–proteasome system (UPS) plays a crucial role not only in protein homeostasis, but in the regulation of the activation or inactivation of signaling molecules involved in cell cycle control, apoptosis, or inflammation [73]. It also shows the importance of this system that around 80% of intracellular proteins are broken down by this mechanism [74].

It is a critical decision to be made which protein(s) should be eliminated: for this purpose, a unique signaling system has evolved in cells using ubiquitin, which is a highly conserved polypeptide consisting of 76 amino acids. Ubiquitin molecules can be covalently attached to lysine (Lys, K) residues in the target proteins [75,76] by an enzyme system consisting of E1 (ubiquitin-activating), E2 (ubiquitin-conjugating), and E3 (ubiquitin ligase) activities [77,78]. Polyubiquitinated proteins are recognized by the 26S proteasome complex, which subsequently breaks them down into small peptides by its proteolytic activity [79]. The E1 enzyme activates the ubiquitin monomer in the presence of ATP by forming a high-energy thioester bond between the C-terminal glycine and the catalytic cysteine of the E1 enzyme [80]. Activated ubiquitin is then transferred to the reactive cysteine side chain of E2 [81]. Finally, the E3 enzyme transfers ubiquitin from E2 to the amino group of the reactive lysine side chain of the substrate protein [82]. Polyubiquitin chains are formed by repeating the previous steps and connecting ubiquitin molecules with isopeptide bonds to each other, which then directs the protein to the proteasome. The specificity of the system is shown by the fact that there are 2 known types of E1, 60 types of E2, and more than 600 different substrate protein-specific enzymes of E3 ubiquitin ligases [82]. This is crucial in the regulation of signaling processes inside cells, because a certain signaling protein can be ubiquitinated only by its corresponding E3, thus ensuring its precisely controlled degradation by the proteasome [83].

One of the best-known E3 ubiquitin ligases is Mdm2 (mouse double minute 2), which can polyubiquitinate the p53 tumor suppressor protein, which step is then followed by the proteasomal degradation [84]. Accordingly, excessive Mdm2 activity favors the development of various tumors [85]. Thus, ubiquitin ligase inhibitors (e.g., Nutlin) are used in the therapy of p53-positive tumors with encouraging results [86]. Another well-known E3 ubiquitin ligase is the SCF^βTrCP^, which plays a crucial role in the polyubiquitination of the inhibitor of nuclear factor-κB (IκB) [87], essential for the activation of nuclear factor-κB (NF-κB), which results, for example, in the production of pro-inflammatory cytokines by immune cells [88].

The 26S proteasome is a cylindrical multicatalytic proteinase complex located in the cytoplasm and nucleus of eukaryotic cells. It consists of a 20S catalytic core, which is organized into two α and two β rings containing seven subunits each (α7β7β7α7), and two 19S regulatory caps. The lid of the 19S cap is responsible for the recognition of ubiquitinated proteins while the base contains ATPases required for the unfolding of proteins, and it also translocates them into the catalytic core. Three subunits of the β rings (β1, β2, β5) are responsible for the proteolytic activities of the proteasome. The β1 subunits possess caspase-like activity and cut the polypeptide chain after acidic amino acid residues, the β2 subunits have trypsin-like activities cleaving after basic residues, and the β5 catalytic subunits show chymotrypsin-like activities severing the polypeptide chain after hydrophobic residues [89]. Each protease can be differentially inhibited by various proteasome inhibitors (PI). Ubiquitin molecules are cut from the protein to be degraded by deubiquitinating (DUB) enzymes. Once a ubiquitin is released, it can be reused and bound to other proteins again [90].

## 3. The Immunoproteasome

Immunoproteasomes are present in cells of hematopoietic origin, especially in lymphocytes and monocytes [91,92], and they are formed by replacing the standard β1, β2, and β5 catalytic subunits with the immunoproteasome subunits β1i, or low-molecular-mass polypeptide-2 (LMP2); β2i, or multicatalytic endopeptidase complex-like-1 (MECL-1); and β5i, or low-molecular-mass polypeptide-7 (LMP7) [93]. The expression of these subunits can also be induced in non-hematopoietic cells after exposure to inflammatory cytokines like IFNγ and TNFα [94,95]. Consequently, the de novo synthesis of the 20S proteasome in inflamed tissues is largely in the form of immunoproteasomes [96]. These exchanges alter the cleavage specificity of the core particle in the immunoproteasome as follows: the caspase-like activity is strongly reduced, whereas the chymotrypsin-like activity is enhanced. In immunoproteasomes, one or both 19S structures are replaced by PA28 as the regulatory cap [97].

Elevated immunoproteasome levels have been associated with inflammation and the development and progression of autoimmunity [98,99]. Immunoproteasome expression can also be detected in tumors to a varying degree, depending on cancer type and differentiation status. Furthermore, mixed structures consisting of standard and immunoproteasome subunits have also been described [100]. Apart from constitutive and immunoproteasomes, a third proteasome variant, designated thymo-proteasome, was identified in cortical thymic epithelial cells. Its function seems to be required for the positive selection of CD8^+^ T cells and in the control of cytokine release [101]. Finally, studies in knockout mice have demonstrated a role for immunoproteasome subunits in generating MHC-I ligands, establishing the naive CD8^+^ T-cell repertoire and shaping cytotoxic T-cell responses [102,103].

## 4. Proteasome Inhibitors (PIs)

Because proteins that regulate the cell cycle, cell proliferation, differentiation, apoptosis, and inflammation are degraded by the UPS, it can influence the activation status of various intracellular signaling pathways [104]. In addition, it also destroys abnormal proteins derived from processes like oxidative stress or mutation, thus preventing them from disturbing normal cellular homeostasis. Inadequate function of the UPS is described in the background of various diseases, such as tumors [105], neurodegeneration [106], or autoimmune/inflammatory diseases [107]. For this reason, targeted inhibition of the UPS appears to be a logical therapeutic approach in such diseases [108]. There are different types of PIs, which can be grouped based on their chemical structure. Peptidyl-aldehyde, peptide-boronate, epoxyketone, and β-lactone type compounds are the best-known categories (Table 1).

The first identified PI was lactacystin, a naturally occurring β-lactone-type compound produced by Streptomyces bacteria, which was noted for its neuritogenesis-inducing effect [109]. Its proteasome inhibitory activity was revealed only later, in 1995 [110]. Its biologically active form, clasto-lactacystin-β-lactone, also known as omuralid, is formed by spontaneous transformation in aqueous solutions at neutral pH [111,112,113]. This derivative then irreversibly inhibits proteasome function as a pseudosubstrate by covalently binding to the hydroxyl groups of threonines in the active center of the β-subunits [110,111,114].

The most studied representative of the peptide-boronates, bortezomib (also known as BTZ, PS-341, Velcade^®^), is among the best-known proteasome inhibitors (Table 1). It was approved by the FDA (Food and Drug Administration) in 2003 for the treatment of multiple myeloma [115]. Since then, it has been used more and more widely, primarily in the therapy of hematological tumors. As peptidomimetics, boronate-type PIs bind reversibly to threonine amino acids in the active center of the proteasome [116]. Multiple myeloma tumor cells are much more sensitive to PI treatment than healthy cells [117], because they are characterized by particularly elevated protein synthesis, and their increased NF-κB activity was also observed compared with that of healthy hematopoietic cells [74].

Rapid protein synthesis can result in the production of unfolded or misfolded proteins, and their elimination by the proteasomes is essential for the homeostasis of cells. At the same time, the activation of NF-κB also requires the function of proteasomes, since in addition to being responsible for the degradation of IκB, an inhibitor of the NF-κB transcription factor [118], the formation of active NF-κB subunits also requires the proteasomal cleavage of larger propeptides [118,119]. Accordingly, when proteasome activity is inhibited, the accumulation of unfolded and misfolded proteins leads to endoplasmic reticulum (ER) stress and unfolded protein response (UPR) in addition to the reduction in NF-κB activity. An approximately 80% decrease in proteasome activity does not affect the function of healthy cells yet, but at the same time, it leads to apoptosis of multiple myeloma cells [74]. Additionally, bortezomib inhibits cytokine production in vitro and inflammation in vivo, too [88,120]. Delanzomib (CEP-18770), which was also tested in a rat collagen-induced arthritis (CIA) model of RA, belongs to this group as well (Table 1). In 2015, another boronate-type PI, Ixazomib (also known as MLN 9708, Ninlaro^®^) [121] was approved by the FDA for the treatment of multiple myeloma. The great advantage of this compound is that it can be taken orally, while the others require intravenous (or possibly subcutaneous) administration [122].

MG-132 (also known as carbobenzoxyl-leucinyl-leucinyl-leucinal, Z-Leu-Leu-Leu-al, Cbz-LLL, or z-LLL) belongs to the synthetic peptidyl-aldehyde type PIs (Table 1) [123]. Penetrating easily through the cell membrane, it can reversibly inhibit primarily the chymotrypsin-like activity of the proteasome [114], thus affecting, among others, the degradation of many signaling proteins [124]. Similar to boronate-type compounds, peptidyl-aldehydes act as peptidomimetics and reversibly inhibit the proteasome activity [123]. In addition, they can also inhibit other proteases (calpains and certain lysosomal cysteine proteases), although only at higher concentrations [114,125].

**Table 1:** Summary of proteasome inhibitors’ (PIs) basic characteristics and their effects tested in RA or related models.

PI	Group	Binding	Subunit Specificity *	Animal Model/Disease	Ref.	Main Findings
**Bortezomib (PS-341, Velcade^®^)**	Peptide- boronates	Rev.	β5/β5i > β1/β1i > β2i	SCWIA ^1^	[88]	(i) total arthritis index and hind paw edema ↓ (ii) inflammatory cell infiltration ↓ (iii) cartilage and subchondral bone degradation ↓ (iv) serum IL-6 and NO metabolites ↓ (v) NF-κB activation ↓ (vi) synovial hyperproliferation ↓ (vii) clonal expansion of T cells ↓
				Zymosan-induced arthritis	[126]	(i) LPS-induced *SAA1* expression (induced by NF-κB) ↓ (ii) zymosan-induced acute arthritis ↓
				CIA	[127]	(i) arthritis score and thickness of paws ↓ (ii) inflammatory cell infiltration ↓ (iii) erosion score ↓ (iv) TNFα, IL-1β, IL-6, MMP-3, iNOS, and COX-2 levels ↓ (v) joint destruction on micro-CT ↓ (vi) no adverse effects
				PBMC of RA patients	[128]	(i) cytokine production (TNFα, IL-1β, IL-6, IL-10 induced by NF-κB) of activated T cells ↓ (ii) T-cell activation ↓ (iii) apoptosis of activated T cells ↑
				AIA	[129]	(i) proliferation of splenocytes and FLS ↓ (ii) FLS invasion ↓ (iii) apoptosis of FLS and splenic T cells ↑ (iv) proliferation ↓↓, apoptosis ↑↑ of activated cells (v) cytokine (IFNγ, TNFα, and IL-6 induced by NF-κB) production ↓ (vi) inflammatory cell infiltration (T cells, B cells, macrophages) ↓ (vii) bone erosion ↓ (viii) pannus formation ↓ (ix) expression of CD3, CD79a, CD11b, COX1, and factor VIII in the joints ↓ (x) expression of TLR-2, -3, -4 in the peripheral blood and cultured FLS ↓ (xi) disappearance of soft tissue swelling, focal osteopenia, and bone erosion on CT images
				hTNFtg	[130]	(i) paw swelling ↑ (ii) grip strength ↓ (iii) area of inflammation ↑ (iv) cartilage proteoglycan loss ↑ (v) T cells ↑, B cells ↓, macrophages ↓ in the synovium (vi) CD8^+^ T cells ↑ in the spleen (vii) no significant change in TNFα, GM-CSF, IFNγ, IL-17, IL-4, and IL-10 in the serum (viii) synovial osteoclastogenesis ↑ (ix) serum levels and synovial expression of RANKL ↑ (x) no effect on the systemic bone architecture and turnover (xi) bone erosion ↑ (xii) osteoclastogenesis ↑, osteoclast apoptosis ↑ at higher cc (xiii) c-Fos and NFATc1 expression ↑
				RA + MM ^†^	[131]	(i) RA activity ↓ (ii) joint symptoms ↓ (iii) DAS28-ESR improved
				RA + MM ^†^	[132]	(i) joint symptoms ↓ (ii) pain ↓ (iii) signs of inflammation ↓
**Delanzomib (CEP-18770)**	Peptide- boronates	Rev.	β5/β5i > β1/β1i	CIA	[133]	(i) severity of arthritis ↓ (ii) TNFα, IL-6, and CRP levels ↓ (iii) elimination of adalimumab ↓ (iv) did not inhibit the production of anti-adalimumab antibodies (v) FcRn levels ↑
**MG-132**	Peptidyl- aldehydes	Rev.	β5/β5i > β1/β1i	SCWIA	[134]	(i) IL-1β, IL-6, TNFα, and VCAM-1 (induced by NF-κB) ↓ (ii) TNFα- and FasL-induced apoptosis in RA synovium ↑ (iii) prevented the development of arthritis
				AIA	[135]	(i) arthritis severity ↓ (ii) pain behavior ↓ (iii) weight loss ↓ (iv) osteoporosis and bone erosion score ↓ (v) inflammatory cell infiltration ↓ (vi) synovial thickening ↓ (vii) no effect on joint space narrowing and cartilage destruction (viii) NF-κB and p50 homodimer DNA-binding activity ↓ (ix) p50-positive cells in the cartilage and in the synovium ↓ (x) sensory neuropeptide (SP, CGRP) production ↓
				RASFs of AIA	[136]	(i) MMP-2 activity ↓ (ii) sFKN production ↓ (iii) TNFα- and IFNγ-induced proteasome activation ↓
				AIA	[137]	(i) SP expression in DRG and in SC ↓ (ii) number of SP-positive cells in the DRG ↓ (iii) NF-κB DNA-binding activity in the SC ↓
**Carfilzomib (PR-171,** **Kyprolis^®^)**	Epoxyketons	Irrev.	β5/β5i >> β2i∼β1i	PBMC of RA patients	[96]	resistance caused by the overexpression of Pgp
**ONX-0914** **(PR-957)**	Epoxyketons	Irrev.	β5i > β1i, β2i	CAIA and CIA	[138]	(i) reversed the signs of disease (ii) cytokine production of T cells (IL-2, IFNγ) and monocytes (IL-23) ↓ (iii) autoantibody levels ↓ (iv) inflammatory infiltration ↓ (v) bone erosion ↓
				PBMC of RA patients	[96]	resistance caused by the overexpression of Pgp
				PBMCs and FLSs of RA patients, CIA	[139]	(i) Th1, Th17↓; Th2, Treg↑ (ii) FLS apoptosis ↑ (iii) FLS invasiveness and viability ↓ (iv) inflammatory markers (IL-6, CCL2, MMP1, MMP3) ↓ (v) arthritis score ↓ (vi) synovial hyperproliferation ↓ (vii) articular injury ↓ (viii) serum TNFα, IL-1β, and IFNγ ↓ (ix) synovial cell apoptosis ↑ (x) BCL2, Vimentin, and VEGF expression in the synovium ↓

^1^ Abbreviations used in the table: SCWIA, streptococcal cell wall-induced polyarthritis in rats; CIA, collagen-induced arthritis; CAIA, collagen antibody-induced arthritis; AIA, adjuvant-induced arthritis; hTNFtg, human TNFα-transgenic mice; PBMCs, peripheral blood mononuclear cells; FLSs, fibroblast-like synoviocytes; RASFs, rheumatoid arthritis synovial fibroblasts; MM, multiple myeloma; SAA1, serum amyloid A1; MMP, matrix metalloproteinase; COX, cyclooxygenase; RANKL, receptor activator of nuclear factor-κB ligand; DAS28-ESR, Disease Activity Score and Erythrocyte Sedimentation Rate; VCAM-1, vascular cell adhesion molecule 1; SP, substance P; CGRP, calcitonin gene-related peptide; DRG, dorsal root ganglia; SC, spinal cord; Pgp, P glycoprotein; CCL2, chemokine (C-C motif) ligand 2; BCL2, B-cell lymphoma 2; VEGF, vascular endothelial growth factor; Rev., reversible; Irrev., irreversible. * Subunit specificities based on [140]. ^†^ Case report. ↓: decreased; ↑: increased

Epoxyketones bind covalently to threonines in the active center of the catalytic β subunits (Table 1) [141,142], so they inhibit irreversibly the function of proteasomes. Epoxomicin was the first representative of this group, which primarily inhibits the chymotrypsin-like activity of the β5 subunit. A 100- to 1000-fold concentration is required to block the activity of the other two catalytic subunits (β1, β2) [141]. The activity of other cellular proteases, such as calpain or cathepsin, is not affected by epoxomicin up to a concentration of 50 µM [141,142]. Garrett and colleagues reported the impact of epoxomicin in bone formation in their in vitro and in vivo experiments [143], which could also be beneficial in the therapy of RA.

In 2012, an agent belonging to this group was also approved by the FDA for the treatment of multiple myeloma. Carfilzomib (also known as PR-171, Kyprolis^®^) [144] was tested in mononuclear blood cells of patients with RA together with another epoxyketone type PI, called ONX-0914 (Table 1) [97].

ONX-0914 (also known as PR-957) is a selective inhibitor of the chymotrypsin-like subunit of the immunoproteasome (β5i = LMP7), which inhibited LMP7 activity by more than 80% with minimal inhibition of the LMP2 or MECL-1 at concentrations less than 100 nM (Table 1). ONX-0914 contains a ketoepoxide pharmacophore that covalently modifies proteasomal N-terminal threonine active sites [138]. It blocked the presentation of LMP7-specific, MHC-I-restricted antigens in vitro and in vivo and decreased the production of interleukin-23 (IL-23) by activated monocytes and that of IFNγ and IL-2 by T cells [138]. However, most well-characterized proteasome inhibitors mediate the equivalent inhibition of both proteasome chymotrypsin-like activities (β5 and β5i = LMP7) [145,146] and have considerable toxicities that probably limit their clinical utility in chronic inflammatory diseases such as RA [147,148]. ONX-0914 inhibits the function of the immunoproteasomes present at the site of inflammation and in immune cells.

## 5. Proteasome Inhibitors in RA or Its Animal Models

As described above, the UPS plays a critical role in pro-inflammatory signaling due to the proteasomal degradation of IκB, which sequesters NF-κB in the cytoplasm, preventing the nuclear translocation of the transcription factor and the subsequent expression of pro-inflammatory cytokines (e.g., TNFα, IL-1β, IL-6, and IL-10) [128]. In addition to these cytokines, NF-κB induces the expression of some anti-apoptotic and osteoclast-activating proteins [34]. The UPS has a role not only in the breakdown of IκB but in the processing of NF-κB precursor molecules to mature p50 and p52 NF-κB subunits [119]. From this perspective, it has been anticipated that inhibitors of the proteasome may counteract the NF-κB activation process and elicit a potential anti-inflammatory response. They may also prevent bone resorption by interfering with the NF-κB-mediated osteoclast activation through the RANKL signaling pathway or by enhancing osteoblast activity [135]. The combined inhibition of more pro-inflammatory cytokines and osteoclast activation by PIs could offer a unique new therapeutic approach in addition to the existing ones. In the following sections we give a brief overview of the results that were published about the effects of PIs in RA or its models (Table 1).

### 5.1. Bortezomib

Not surprisingly, since bortezomib was the first FDA-approved PI, this chemical was the most studied also in autoimmune arthritis (Table 1). Palombella and colleagues [88] investigated the effect of bortezomib in the Streptococcal cell wall (SCW)-induced rat model of RA (Table 1). They found that the daily administration of bortezomib orally, beginning 7 days after the induction of arthritis, significantly attenuated the progression of chronic polyarthritis as shown by the decreased total arthritis index and hind paw edema. In the used dose, bortezomib reduced proteasome activity around 55% in circulating leukocytes 4 h after the treatment. Histological sections proved diminished inflammatory cell infiltration and reduced cartilage and subchondral bone degradation in the PI-treated rats. The pannus and the thickened synovial membrane exhibited a marked reduction in inflammatory cell infiltration due to the bortezomib treatment. Serum IL-6 and nitrogen monoxide (NO) metabolites were reduced; however, serum IL-1 levels remained high in this animal model after bortezomib treatment. Bortezomib inhibits the production of proinflammatory mediators by blocking the degradation of IκB and the subsequent activation of NF-κB. Moreover, it can also prevent cell cycle progression by blocking the degradation of key regulatory proteins, which can reduce synovial hyperproliferation and the clonal expansion of T cells underlying the progression of RA.

In another study, Zhang and colleagues reported their results from experiments using a serum amyloid A1 (SAA1)-luciferase (*Saa1-luc*) transgenic mouse model in which the SAA1 gene expression can be monitored by measuring luciferase activity using a noninvasive imaging system (Table 1) [126]. The acute-phase serum amyloid A proteins are produced in large quantities during the acute phase of inflammation and during the development of chronic inflammatory diseases like, for example, RA [126]. On the other hand, the *SAA1* promoter has an NF-κB-responsive element, meaning that *SAA1* expression can be regulated by the NF-κB transcription factor [126]. The authors showed that *SAA1* promoter-driven luciferase expression could be induced by LPS, TNFα, or IL-1β, and the increased luciferase activity (1000- to 3000-fold) could be detected in the hepatic region of *Saa1-luc* mice [126]. Pretreatment with bortezomib (1 mg/kg i.v. 1 h before LPS treatment) suppressed LPS-induced luciferase activity by 90% [126]. These results suggested that proteasome inhibition may regulate *SAA1* expression, perhaps through the inhibition of the NF-κB pathway [131]. During induction of acute arthritis by intra-articular administration of zymosan, *SAA1* promoter-driven luciferase expression was induced both locally at the knee joint and systemically in the liver, and the induction was significantly reduced by bortezomib in both locations [126].

In collagen-induced arthritis (CIA), significant improvement in arthritis severity (as shown by a decrease in the arthritis score and the thickness of the paws) was found after bortezomib treatment (Table 1) [127]. Histological examinations showed a significant decrease in inflammatory cell infiltration, erosion score, TNFα, IL-1β, IL-6, matrix metalloproteinase-3 (MMP-3), inducible nitric oxide synthase (iNOS), and cyclooxygenase-2 (COX-2) levels upon bortezomib treatment compared with the untreated CIA mice [127]. In addition, micro-CT confirmed that bortezomib reduced joint destruction [127]. No adverse effects were observed in the blood cells, liver, or kidneys caused by bortezomib treatment [127]. The authors conclude that their data suggest that bortezomib can have an anti-inflammatory role in the pathophysiology of RA and that it may serve as a new therapeutic approach for the disease.

In a study by Van der Heijden and colleagues, whole blood samples of 19 RA patients and 7 healthy controls were examined ex vivo (Table 1) [128]. The effects of bortezomib on cytokine production, T-cell apoptosis, and T-cell activation was tested after T-cell activation with anti-CD3/CD28. Bortezomib inhibited the cytokine (TNFα, IL-1β, IL-6, IL-10) production of activated T cells in the RA and the control samples [128]. IL-10 production was less efficiently blocked in RA patients compared with healthy controls. They investigated the baseline TNFα production of activated T cells and the effects of bortezomib and methotrexate on this process [128]. Following anti-CD3/CD28 stimulation of T cells, they could not find a significant difference in the TNFα production in whole blood incubates from healthy volunteers and RA patients [128]. The median concentration of bortezomib required to inhibit TNFα production by 50% (IC50) was significantly higher in the RA patients than the healthy controls, while there was no significant difference between the IC50 of methotrexate [128]. At a later stage of bortezomib treatment (48 h), significant apoptosis induction was observed in peripheral blood lymphocytes (PBLs) of RA patients when compared with the healthy controls, while there was no such difference after 24 h of treatment [128]. Methotrexate, at the same time, did not induce apoptosis in these cells even after 48 h [128]. The authors found a decreased CD25 expression in the same set of cells after 48 h exposure to bortezomib, which inhibitory effect of the drug on T-cell activation was not significant after 24 h of treatment [128].

Significant attenuation of adjuvant-induced arthritis (AIA) in rats was reported as a result of bortezomib treatment (Table 1) [129]. In in vitro experiments, bortezomib inhibited the proliferation of splenocytes and fibroblast-like synoviocytes (FLS), reduced FLS invasion, and induced apoptosis of FLS and splenic T lymphocytes. The activated cells showed a more pronounced reduction in cell proliferation and were more susceptible to bortezomib-induced cell death. In the case of FLS, 10 nM bortezomib reduced their invasion capacity, while at a 50 nM concentration, apoptosis was observed in these cells, which have a critical role in the initiation as well as the progression of RA. Bortezomib down-regulated the cytokine (IFNγ, TNFα, and IL-6) production of the cultured splenocytes. After i.p. administration of bortezomib at a concentration of 0.25 mg/kg (twice weekly for 2 weeks), treated animals exhibited reduced disease severity. In histology sections, the authors found decreased inflammatory infiltration (T and B lymphocytes, macrophages), little or no bone erosion, and limited pannus formation in the joints of the bortezomib-treated rats. They could identify decreased expressions of CD3, CD79a, CD11b, COX1, and factor VIII in the joints and of TLR-2, -3, and -4 in the peripheral blood and cultured FLS in the bortezomib-treated rats with AIA. Additionally, CT imaging also proved the attenuation of AIA after bortezomib treatment, namely, the disappearance of soft-tissue swelling, focal osteopenia, and bone erosion.

Bortezomib treatment was investigated in a mouse model of inflammatory arthritis in which they used human TNFα transgenic (hTNFtg) mice (Table 1) [130]. Surprisingly, they found moderately increased inflammatory activity after bortezomib treatment in this model of arthritis. Although they measured more pronounced paw swelling and loss of grip strength in the treated animals, the difference did not reach statistical significance. Bortezomib-treated hTNFtg mice showed a slight increase in the area of inflammation, more severe cartilage proteoglycan loss, and quantitatively as well as qualitatively altered inflammatory infiltration compared with the vehicle-treated control group. They identified more T cells and fewer macrophages and B cells in the synovium of the bortezomib-treated mice, while in the spleens of the same mice, only the CD8^+^ T cell number increased; the B-cell, macrophage, Treg, and CD4^+^ T-cell numbers remained unchanged. Additionally, they could not detect any significant change in the serum concentrations of TNFα, GM-CSF, IFNγ, IL-17, IL-4, and IL-10 after bortezomib treatment of hTNFtg mice. Contrary to other published data, Polzer and colleagues stated that bortezomib worsens the TNF-mediated arthritic bone destruction associated with increased synovial osteoclastogenesis [130]. Subsequently the authors showed increased serum levels and synovial expression of RANKL due to bortezomib treatment, which could contribute to the elevated synovial osteoclastogenesis and joint damage they found [130]. At the same time, the serum levels and synovial expression of osteoprotegerin (OPG) did not change significantly in the same experimental setup. Bortezomib had no effect on the systemic bone architecture and turnover in this model. The latter was indicated by the unchanged levels of serum type I collagen cleavage products due to the treatment. On the other hand, in the presence of synovial inflammation, bortezomib dramatically increased local bone erosions, and in in vitro experiments, the agent showed a dose-dependent stimulatory effect on osteoclastogenesis, while at higher concentrations it induced the apoptosis of osteoclasts [130]. When the authors tested the expression of osteoclast-associated genes, they found elevated mRNA levels of c-Fos and nuclear factor of activated T cells cytoplasmic 1 (NFATc1), which are crucial for osteoclast differentiation, after the treatment with the osteoclast-stimulatory concentrations of bortezomib [130].

Although in scarce numbers, some human data can also be found about the possible effect of bortezomib in RA patients (Table 1). In a case study by Liu and colleagues, a refractory RA patient, who failed methotrexate treatment combined with biologic agents, was diagnosed with MM [131]. After the diagnosis of MM, drugs for RA were discontinued and a bortezomib-based chemotherapy was started. After the first cycle, the symptoms of RA alleviated significantly [131]. RA activity decreased, joint symptoms resolved, and both Disease Activity Score and Erythrocyte Sedimentation Rate (DAS28-ESR) improved [131]. In another report, three cases of therapy-resistant RA patients with subsequently diagnosed multiple myeloma (MM) were described [132]. Three months after the initiation of bortezomib therapy, the joint manifestations of RA diminished in these cases as well, the pain and signs of inflammation became reduced, and both diseases seemed to be in remission even a couple of months after stopping bortezomib treatment [132].

### 5.2. Delanzomib

Recently, the anti-arthritic effect of delanzomib, a novel boronate-type proteasome inhibitor, was investigated alone or in combination with adalimumab in the CIA rat model of RA (Table 1) [133]. Adalimumab is widely used in the treatment of RA as a biological anti-TNFα therapy. Delanzomib decreased the severity of arthritis and reduced the levels of TNFα, IL-6, and CRP in rats with CIA [133]. However, delanzomib alone had a weaker effect on the improvement of the disease compared with adalimumab alone or when the two medications were combined [133]. The most impressive effect was achieved with the combination therapy, most likely because both drugs contribute to the reduced TNFα levels in vivo [133]. Importantly, delanzomib slowed down the elimination of adalimumab, which could prolong its effect as well. Although delanzomib did not inhibit the production of anti-adalimumab antibodies, it increased the level of neonatal Fc receptors (FcRn), probably by suppressing their degradation, and this could contribute to the reduced elimination of adalimumab [133]. Based on these observations, the authors proposed delanzomib in combination with adalimumab as a potential therapeutic approach for RA [133].

### 5.3. MG-132

The role of NF-κB was investigated in the arthritic synovium of rats with streptococcal cell wall (SCW)-induced arthritis (Table 1) [134]. In vitro experiments with primary synovial fibroblasts indicated the role of NF-κB activation in the induction of IL-1β, IL-6, and TNFα and in the VCAM-1-mediated recruitment of inflammatory cells to RA joints [134]. In addition, they reported the protective effect of NF-κB activation against TNFα- and FasL-induced apoptosis in the RA synovium [134]. In in vivo experiments, they found that NF-κB activation in the synovium of arthritic rats was similar to that in human RA [134]. The inhibition of NF-κB by the intraarticular (i.a.) administration of the proteasome inhibitor MG-132 or by i.a. adenoviral gene transfer of super-repressor IκBα (srIκBα)—which cannot be phosphorylated and degraded—significantly increased apoptosis in the synovium of SCW-induced arthritic rats [134]. These data suggested the anti-apoptotic role of NF-κB activation due to inflammation and hyperplasia in the synovium in RA joints [134]. The i.a. injection of liposomal complexes containing NF-κB decoys 24 h before reactivation of SCW-induced arthritis prevented the development of arthritis unexpectedly, even in the contralateral, untreated joint of the same animal [134].

The effect of MG-132 was also characterized in a rat AIA model (Table 1) [135]. In this report, the research group found a reduction in arthritis severity, pain behavior, weight loss, osteoporosis and bone erosion score, inflammatory cell infiltration, and synovial thickening in arthritic rats treated with MG-132 compared with the vehicle-treated controls [135]. However, the treatment did not affect the narrowing of the joint space or cartilage destruction in the MG-132-treated animals [135]. Importantly, MG-132 significantly decreased the DNA-binding activity of NF-κB and the p50 homodimer in the inflamed ankle as well as the number of p50-positive cells in the cartilage and in the synovium of arthritic rats [135]. On the other hand, MG-132 normalized the elevated expression level of sensory neuropeptides (substance P and calcitonin gene-related peptide) found in arthritic animals [135]. The authors conclude that the therapeutic effect of MG-132 was mediated mainly by the inhibition of NF-κB activation but also involved the peripheral nervous system, and they mentioned new-generation PIs as potential novel pharmacotherapy for RA.

In a continuation of the above work, the authors investigated the effects of MG-132 on the expression of substance P in dorsal root ganglia (DRG) and in the spinal cord (SC) as well as the DNA-binding activity of NF-κB in the SC in a rat AIA model (Table 1) [137]. They found significantly higher substance P expression in the DRG and in the SC of arthritic rats compared with the healthy controls, both of which were significantly decreased by MG-132 treatment; additionally, the number of substance P-positive cells in the DRG also decreased [137]. An increase in the DNA-binding activity of NF-κB was observed in the SC extract of arthritic animals, which was reduced by the MG-132 treatment [137]. The above-described changes were exclusive in arthritic rats, while the MG-132 treatment caused no significant effects in the non-arthritic controls; therefore, MG-132 treatment specifically prevented the molecular changes caused by arthritis [137].

Finally, Jones and colleagues reported in their study with AIA rats that MG-132 inhibited the MMP-2 activity induced by the combination of TNFα and IFNγ in rheumatoid arthritis synovial fibroblasts (RASFs) (Table 1) [136]. Pretreatment of RASFs with the proteasome inhibitor MG-132 completely blocked their TNFα/IFNγ-induced soluble Fractalkine (sFKN) production [136]. Fractalkine, or CX_3_CL1, is a chemokine to which pathological significance was attributed in the development of RA [149]. It has a membrane-bound form (referred to as FKN) that functions as an adhesion molecule for CX_3_CR1-expressing leukocytes and a soluble form (sFKN) that is functioning as a chemoattractant. The 20S proteasome activity of the RASFs was only modestly induced by either TNFα or IFNγ, but this became significantly more prominent when they were present in combination, which could be completely abolished by MG-132 [136].

### 5.4. Carfilzomib

The potential of epoxyketone-based PIs (carfilzomib, ONX-0912, ONX-0914) were investigated as to whether they might overcome two known mechanisms of bortezomib resistance: first, the mutation of the β5 (*PSMB5*) subunit of the proteasome, and second, the drug efflux mediated by the P-glycoprotein (Pgp)/multidrug resistance 1 (MDR1)/ATP-binding cassette B1(ABCB1) (Table 1) [96]. Peripheral blood mononuclear cells (PBMCs) from therapy-naive RA patients showed low Pgp levels ex vivo; however, its expression could compromise the inhibitory effect of carfilzomib and ONX-0914, which could be significantly reduced by a Pgp transport inhibitor P121 (reversin 121) [96].

### 5.5. ONX-0914

Muchamuel and colleagues [138] investigated ONX-0914, an immunoproteasome inhibitor specific for the β5i (=LMP7), which blocks the presentation of LMP7-specific, MHC-I-restricted antigens in vitro and in vivo (Table 1). ONX-0914 at concentrations less than 100 nM inhibited LMP7 activity by more than 80% with minimal inhibition of LMP2 or MECL-1, the other two immunoproteasome catalytic subunits. According to their results, ONX-0914 blocked the production of IL-23 by activated monocytes and that of IFNγ and IL-2 by T cells. In mouse models of rheumatoid arthritis, collagen antibody-induced arthritis (CAIA), and CIA, ONX-0914 treatment reversed the signs of disease and resulted in reductions in cellular infiltration, cytokine production, and autoantibody levels.

In the study by Liu and colleagues, PBMCs and FLSs from RA patients and the CIA mouse model of the disease were investigated (Table 1) [139]. They found that LMP7 expression was higher in RA patients than in healthy controls, and this expression was positively correlated with Th1 cells, the Th1/Th2 ratio, Th17 cells, and the Th17/Treg ratio but not with Th2 or Treg cells [139]. The immunoproteasome inhibitor ONX-0914 reduced the Th1 and Th17 differentiation of RA-CD4^+^ T cells but promoted their differentiation in the Th2 and Treg direction, which is beneficial in cases of RA [139]. When co-cultured with RA-CD4^+^ T cells, ONX-0914 increased RA-FLS apoptosis and decreased their invasive ability, viability, and inflammation (as suggested by decreased IL-6, CCL2, MMP1, and MMP3 levels), although it had a lesser impact on the RA-FLSs when they were without CD4^+^ T cells in the co-culture [139]. In CIA mice, the LMP expression was decreased in the synovium and in CD4^+^ T cells upon ONX-0914 treatment, and at the same time, it decreased the arthritis score, synovial hyperproliferation, and articular injury [139]. The inflammatory markers IL-6, CCL2, MMP1, and MMP3 in the synovium and TNFα, IL-1β, and IFNγ in the serum of mice were also reduced by ONX-0914 treatment. On the other hand, the imbalance of Th1/Th2 and Th17/Treg in the spleen was significantly decreased, characterized by reduced Th1 and Th17 and enhanced Th2 and Treg differentiation due to the treatment [139]. Moreover, ONX-0914 treatment induced elevated synovial cell apoptosis and reduced BCL2, Vimentin, and VEGF expression in the synovium [139]. As a conclusion, they claim that ONX-0914 treatment ameliorates RA progression and inflammation by repressing LMP7-mediated CD4^+^ T cell imbalance [139].

## 6. Perspectives

Since RA is a chronic and progressive disease, it usually needs long-term therapy after the diagnosis. The ideal therapy would prevent the progression of the disease and create a symptom-free state for a long time by inhibiting the molecular mechanisms involved in the development of RA while not affecting the physiological functions of normal cells. Such a treatment does not exist so far. All currently available treatments have serious side effects, and some patients develop resistance against them. Therefore, it is necessary to search for new therapeutic agents.

One group of candidates could be the PIs, which were originally designed to treat cachexia in end-stage tumor cases, but it turned out within a short time that they have beneficial effects in the treatment of hematological malignancies, in particular, the myeloma multiplex. Later, their use arose in autoimmune and auto-inflammatory diseases as well, which is proved by accumulating data. In autoimmune diseases, we can find the over-activation of immune cells and the decreased apoptosis or increased cytokine production of activated immune cells, which could be successfully regulated by PI treatment. Conventional PIs have beneficial effects in cases of autoimmune diseases like RA, but they can have more side effects because they inhibit the proteasomal function in all kinds of cells. On the other hand, the use of immunoproteasome inhibitors might reduce the proteolytic activity of proteasomes selectively in the immune cells involved in inflammation, which possibly would lead to fewer side effects.

Currently, there are three publicly available clinical trials of PIs in connection with RA treatment at clinicaltrials.gov (accessed on 10 February 2025). All of them investigate(d) the effects of bortezomib. The first study, called “Therapy of Antibody-mediated Autoimmune Diseases by Bortezomib” (TAVAB), started in 2014 in Germany, but they were not able to recruit appropriate RA patients, and the study was terminated in 2019 [43]. The two other studies were started in Beijing, China in 2023, and they want to answer the following questions: (1) Is bortezomib an effective treatment option for patients with difficult-to-treat rheumatoid arthritis? (2) Is bortezomib safe enough in treating patients with difficult-to-treat rheumatoid arthritis? One of them is recruiting patients at present, while the other is active but not recruiting.

Based on the features described above, we propose that PIs could gain a place in the therapeutic spectrum for RA, especially in patients who responded poorly to other available therapies. PIs could be used alone, in combination, or alternating with other treatment modalities. The investigation of PIs beyond bortezomib seems logical and promising for RA therapy. Of course, more PIs should be tested in preclinical (animal) RA models before taking them on to clinical trials.

## Data Availability

The original contributions presented in the study are included in the article; further inquiries can be directed to the corresponding author.
